# Supplementary material for: The interactome of intact mitochondria by cross-linking mass spectrometry provides evidence for coexisting respiratory supercomplexes
Source: Mol Cell Proteomics. 2017 Dec 8;17(2):216–32. doi: 10.1074/mcp.RA117.000470 (PMC5795388; doi:10.1074/mcp.RA117.000470)
Supplement: Supplemental Data [file supp_RA117.000470_133922_0_supp_23978_fzffwf.zip › spectra_annotation/mito_spectra_annotation/73-1-5-1-16-1.pdf]

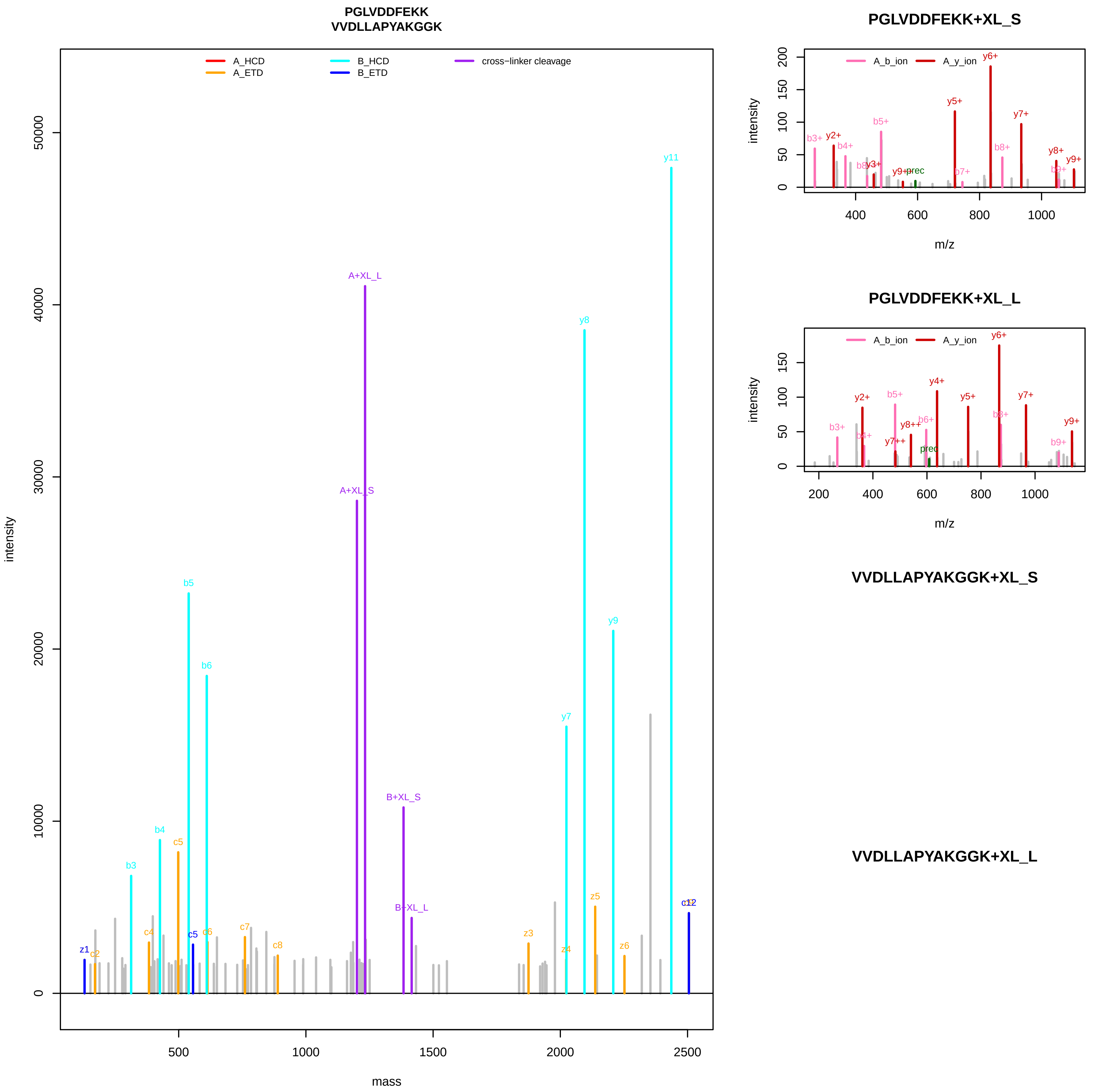
PGLVDDFEKK
VVDLLAPYAKGGK
A_HCD
A_ETD
B_HCD
B_ETD
cross−linker cleavage
intensity
mass
0
10000
20000
30000
40000
50000
500
1000
1500
2000
2500
z1
c2
b3
c4
b4
c5
b5
c5
c6
b6
c7
c8
A+XL_S
A+XL_L
B+XL_S
B+XL_L
z3
y7
z4
y8
z5
y9
z6
y11
c12
PGLVDDFEKK+XL_S
A_b_ion
A_y_ion
b3+
y2+
b4+
b5+
y3+
y9++
prec
y5+
b7+
y6+
b8+
y7+
y8+
b9+
y9+
400
600
800
1000
0
50
100
150
200
m/z
PGLVDDFEKK+XL_L
A_b_ion
A_y_ion
b3+
b4+
y2+
b5+
y7++
y8++
b6+
prec
y4+
y5+
y6+
b8+
y7+
b9+
y9+
200
400
600
800
1000
0
50
100
150
m/z
VVDLLAPYAKGGK+XL_S
VVDLLAPYAKGGK+XL_L
